# Cholinergic Pairing with Visual Activation Results in Long-Term Enhancement of Visual Evoked Potentials

**DOI:** 10.1371/journal.pone.0005995

**Published:** 2009-06-22

**Authors:** Jun Il Kang, Elvire Vaucher

**Affiliations:** Ecole d'optométrie, Université de Montréal, Montréal, Québec, Canada; National Microelectronics Center, Spain

## Abstract

Acetylcholine (ACh) contributes to learning processes by modulating cortical plasticity in terms of intensity of neuronal activity and selectivity properties of cortical neurons. However, it is not known if ACh induces long term effects within the primary visual cortex (V1) that could sustain visual learning mechanisms. In the present study we analyzed visual evoked potentials (VEPs) in V1 of rats during a 4–8 h period after coupling visual stimulation to an intracortical injection of ACh analog carbachol or stimulation of basal forebrain. To clarify the action of ACh on VEP activity in V1, we individually pre-injected muscarinic (scopolamine), nicotinic (mecamylamine), α7 (methyllycaconitine), and NMDA (CPP) receptor antagonists before carbachol infusion. Stimulation of the cholinergic system paired with visual stimulation significantly increased VEP amplitude (56%) during a 6 h period. Pre-treatment with scopolamine, mecamylamine and CPP completely abolished this long-term enhancement, while α7 inhibition induced an instant increase of VEP amplitude. This suggests a role of ACh in facilitating visual stimuli responsiveness through mechanisms comparable to LTP which involve nicotinic and muscarinic receptors with an interaction of NMDA transmission in the visual cortex.

## Introduction

Modulation of visual responses in the primary visual area (V1) by acetylcholine (ACh) contributes to visual attention [1] and learning [2]. In V1, ACh augments cortical plasticity in terms of intensity of neuronal activity [2], [3], [4], [5], [6], [7], [8], preferred responses of visual neurons [6], [9], receptive field properties [6], [10] and performance in visual learning in the visual water maze [2]. Neuronal effects of ACh vary from activation to inhibition [6], [11] depending on the type of muscarinic or nicotinic cholinergic receptors (mAChR and nAChR) activated and location. Overall, the majority of anatomical and physiological data in V1 to date suggests that ACh primarily enhances thalamocortical inputs through the α4β2 nAChR located on the thalamocortical fibres and M1 mAChRs on glutamatergic cells of layer IV [6], [7], [12]. Alternatively, ACh has been shown to decrease the strength of corticocortical input through M2 and M4 mAChRs located on corticocortical fibres [7], [13]. ACh interaction with GABAergic interneurons through α7 nAChRs [14], [15] also contributes to the modulation of sensory responses. The rapid desensitization and high calcium permeability properties of α7 nAChRs could also play a key role in cortical synaptic plasticity, although this action has not been investigated in V1 [16], [17].

Long-term modification of cortical responsiveness such as long-term potentiation (LTP) or depression (LTD) has been proposed as a necessary correlate of learning. The cholinergic system has been shown to enhance long-term activation in certain cortical areas [8], [10]. Repetitive pairing of cholinergic and auditory stimulation over a period of two weeks results in long-term cortical map reorganization [18]. Furthermore, pairing cholinergic activation with somatosensory stimulation [19] induces a long-term (≥1 h) increase of cortical electrophysiological responses. The involvement of ACh in pure LTP or LTD mechanisms, which involves NMDA receptors (NMDAR), has also been demonstrated in the hippocampus and cortex, including V1. Electrophysiologically induced LTP [20], [21] or LTD [22], [23] in V1 or V1 slices [4] is dependent on a cholinergic component. Moreover, LTP and LTD are diminished in V1 of M2/M4 and M1/M3 double knock out mice, respectively [24]. This further indicates a role for ACh in cortical synaptic plasticity through an integrated action of different mAChR subtypes.

These data suggest that ACh may contribute to cortical LTP in V1, similar to other cortical areas [18], [19]. The present study was designed to test the hypothesis that pairing of external stimuli with cholinergic activation induces a long-term enhancement of integrated cortical responsiveness in V1. For this purpose, visual evoked field potentials (VEP) were measured over the course of 4–8 h in V1 after a transient pairing of patterned visual stimulation with local administration of the ACh analog carbachol (CCh) or electrical stimulation of the cholinergic projections to V1. In an attempt to clarify the underlying mechanisms and a possible link with classical LTP mechanisms, the involvement of mAChRs, nAChRs or NMDARs in these responses were tested using scopolamine (a non-selective mAChR antagonist), mecamylamine (non-selective nicotinic receptors antagonist), or -3-(2-carboxypiperazin-4-yl)-propyl-L-phosphonic acid (CPP, NMDAR antagonist). Moreover, the specific role of α7R was tested using methyllycaconitine (MLA, a α7 nAChR selective antagonist) to evaluate the influence of this receptor which has recently been recognized for its involvement in cortical plasticity [14], [17].

## Materials and Methods

### Animal preparation

Adult Long-Evans rats (n = 60, 250–300 g) were obtained from Charles River Canada (St-Constant, Quebec, Canada) and maintained in a 12 h light/dark cycle with free access of food during both the pre- and post-implantation period. Two sets of experiments were performed to evaluate the long-term effects of cholinergic activation paired with visual stimulation on VEPs, i.e. the effects sustained more than 1 h following transient cholinergic stimulation. First, CCh intracortical (i.c.) injections (n = 10) were compared to vehicle injections (n = 11) in order to establish the effects of cholinergic activation on VEPs in V1. To verify the extent of the long-term effects of CCh, 3 animals were tested for an 8 h period. To verify that CCh intracortical infusion mimicked the activation of cholinergic basalo-cortical projections, an electrical stimulation [25] of the V1 projecting cholinergic neurons from the horizontal limb of the diagonal band of Broca (HDB) was performed on another set of animals (n = 4). Second, CCh was used to elucidate the receptors involved in this process. For this purpose 5 different groups in which the following antagonists were injected 1 h prior to CCh were examined: scopolamine (Sco+CCh, n = 4), mecamylamine (Mec+CCh, n = 5), MLA+CCh (n = 6), CPP+CCh (n = 6) and the control group, aCSF+CCh (n = 8). Complementary experiments to better evaluate the involvement of muscarinic receptors included a group of scopolamine i.p. injection 30 min before CCh (Sco i.p.+CCh, n = 5) or simultaneously with CCh (CCh+Sco i.p., n = 2, control group). The antagonistic effect of scopolamine occurs 30 min after it is injected i.p. and persists for around 120 min [26]. These two groups corresponded to inhibition of brain mAChR at the time of or just following CCh injection, respectively. Guidelines set out by the Canadian Council for the Protection of Animals were followed for all procedures and approved by the local Animal Care Committee, “Comité de Déontologie de l'Expérimentation sur les Animaux” at the University of Montreal.

### Surgery

**Figure 1:**
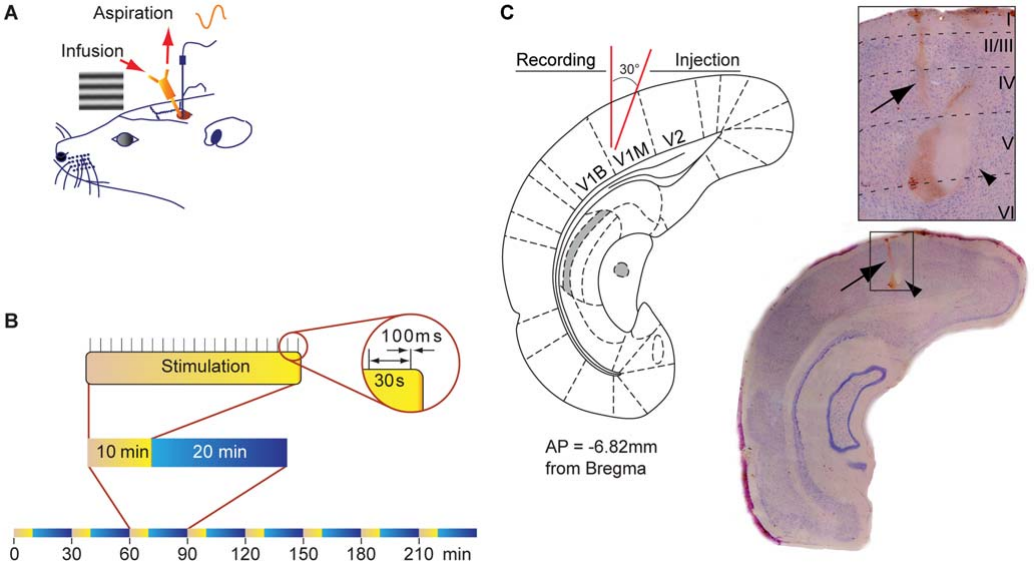
Design of the experiment. (A) Schematic diagram illustrating the chronic implantation of the recoding electrode in V1 and the push-pull cannula guide as well as the lateral stimulation of the retina with a horizontal grating displayed on a computer screen. The push-pull cannula guide and the recording electrode guide were implanted in visual cortex 2 days before VEP recording. (B) Visual stimulation. Rats were stimulated by displaying trains of sinusoidal horizontal grating (100 ms, 0.033 Hz, contrast 100%) for 8 cycles. Each cycle consisted of 10 min visual stimulation every 30 min. The VEP was obtained by averaging the 20 single electrophysiological signals evoked by the 20 presentations of the grating during the stimulation period. (C) Histology of the injection and recording sites. Schematic coronal section at the site of recording and cresyl violet-stained coronal section showing electrolytic lesion indicating position of electrode tip (arrow) and location of the infusion cannula (arrow head). Electrode and cannula tips are adjacent.

Animals were anaesthetized with isoflurane (induction 5%, maintain 1.5%) and placed in a stereotaxic apparatus. Throughout the experiment, the rectal temperature was maintained at 37°C using a thermostatically controlled heating pad (FHC, Bowdoinham, ME, USA). A dental drill was used to make a hole (3.0 mm diameter) in the skull above the left visual cortex. A tungsten electrode (conductance <0.8 MΩ; FHC, Bowdoinham, ME) along with an electrode guide (polyurethane tubing) was then inserted in V1 (mm from Bregma: AP−7.5, ML+4.0, DV−0.5 from dura mater surface) and tested for VEP response. The electrode was removed but the electrode guide was left in place at the surface of the skull. A push-pull cannula guide (Plastics1, Roanoke, VA) was placed adjacent to the electrode tip (mm from Bregma: AP−7.5, ML+3.6, DV−0.7 mm, 30° angle from verticality) (Fig. 1). The stimulating tungsten electrode, denuded at each tip, was implanted in the HDB ipsilateral to the recording cortical site (mm from Bregma: AP−0.3, L+2.0, DV−9.0). Two nylon screws (Small parts, Miami Lakes, FL, USA) were screwed into the skull, then the guides and the HDB implanted electrode were secured with dental cement. After suturing the incised skin, local anaesthesia (xylocaine 2%, Astra Zeneca, Mississauga, Canada) was topically administered to the wound and animals were returned to their cages.

### Drug infusion

All drugs were obtained from Sigma Chemical Co and dissolved in a freshly made artificial cerebrospinal fluid (aCSF: NaCl, 1.0 M; NaHCO_3_, 0.5 M; KCl, 1.47 M; MgSO_4_, 1.25 M; KH_2_PO_4_, 0.25 M; C_6_H_12_O_6_, 0.01 M; CaCl_2_, 1.73 M pH 7.4). Drugs (CCh, 5 mM; scopolamine, 3 µM; mecamylamine, 10 µM; CPP, 20 µM; MLA, 50 nM) or vehicle (aCSF) were injected once intracortically (i.c., 1 µl/min, 10 min, simultaneously to one session of VEP recording) using an injection pump (Harvard Apparatus, Holliston, MA, USA). The push-pull cannula allowed for excess fluids at the injection site to be discarded and limited the accumulation of the drug within the cortex. Intraperitoneal injection of scopolamine (i.p., 10 mg/kg) simultaneously or 30 min prior CCh injection was also performed to compare i.p and i.c. injection regimens and match previous experiments [21], [27].

### HDB electrical stimulation

Electrical stimulation was performed over 10 min period using pulses (100 Hz, 0.5 ms, 50 µA, 1 sec on/1 sec off) generated (Pulsemaster A300, WPI, Sarasota, FL) and delivered through an isolation unit (WPI 365, WPI, Sarasota, FL) [25].

### Visual stimulation paradigm

VEPs were elicited by a patterned visual stimulation provided by trains of sinusoidal gratings displayed on a computer screen in the dark. The computer monitor (30×25 cm, Titanium; luminance 21 cd/m^2^; Apple Computer Inc., Cupertino, CA, USA) was placed parallel to the midline of the rat at a distance of 30 cm [28], [29]. Trains (100 ms on/30 sec off, 10 min) of horizontal sinusoidal grating (contrast 100%, 0.12 cycle/deg) were produced by Vpixx software (v 8.5; Sentinel Medical Research Corp., Quebec, Canada). Selected orientation and spatial frequency of the grating were based on previous studies [27], [28], [29]. Between each grating and during the rest period, the computer screen displayed a neutral grey stimulus with the same mean luminance as the gratings.

### Visual evoked potentials recording procedure

Two days after implantation, rats were placed in the stereotaxic frame under anaesthesia (isoflurane, 1.5%) for VEP recording. The polyurethane tubing (electrode guide) was removed, leaving a hole through the dental cement over V1 through which the electrode was inserted. The electrode was placed 0.5 mm below the dura mater. The penetration of the electrode through the dura mater was identified by the 50% reduction of the mean amplitude of the noise signal without visual stimulation monitored by the audio monitor (AM10, Grass Technologies, Astro-Med, West Warwick, RI, USA) and data acquisition program. The cannula was also inserted within the cortex through the implanted guide. VEPs were calculated by averaging 20 electrical responses of extracellular field potentials over the 10 min stimulation period (trains of 100 msec visual stimuli, 0.03 Hz, Fig. 1). Evoked responses were amplified (5000×) and filtered at 3 Hz∼1 kHz (Grass Inc, West Warwick, RI, USA) and collected with the data acquisition system MP100 and Acknowledge software (v 3.8; Biopac system Inc, Goleta, CA, USA). The amplitude (difference between negative peak and positive peak) and latency (time spent between the artefact of stimulation and the first negative peak) of the VEPs were calculated using this software.

Repetitions of VEP recording were performed every 30 minutes during a 4 h period (Fig. 1). To verify the extent of the long-term effects of CCh, 3 additional animals were tested for an additional 4 h period with the same frequency of VEP recording (sixteen repetitions of VEP recording per animal). Sequence of drug injections were as follows: 1) two baseline VEPs were obtained; 2) then antagonists were injected during the next VEP recording session; 3) then one further VEP was recorded to verify that antagonists or vehicle had no effect by themselves on VEP amplitude; 4) then CCh was injected during the next VEP recording and VEP were recorded for 4 additional periods.

### Histology

At the end of the experiment, an electrolytic lesion was performed to verify the recording site. The animal was then sacrificed by the administration of pentobarbital (30 mg/kg i.p.), the brain was removed, frozen at −50°C in isopentane, and sectioned at 20 µm through the visual cortex using a cryostat (Microm, ESBE, Markham, ON). The sections were then stained with cresyl violet and electrode placement verified.

### Statistical analysis

All quantitative data and the significance of difference in the amplitude of the VEPs between each group and each time point were tested by a mixed model ANOVA with a repeated factor (time) and a non-repeated factor (group). The mixed model ANOVA was used for the 2 sets of experiments, i.e. 4 groups (control, CCh, HDB stimulation, Sco(i.p.)+CCh), or 5 groups (Mec+CCh, MLA+CCh, CPP+CCh, Sco+CCh and aCSF+CCh). In case of a significant (p<0.05) interaction between these factors, a one-way ANOVA followed by the post-hoc LSD test was performed for each time point in order to evaluate drug effects. The same analytical method was applied for the latency. All statistical analyses were carried out with SPSS 16.0 for Windows XP (SPSS Inc., Chicago, IL, USA) with a significance level of p<0.05.

## Results

### Cholinergic stimulation induces a long-term increase of VEP amplitude

**Figure 2:**
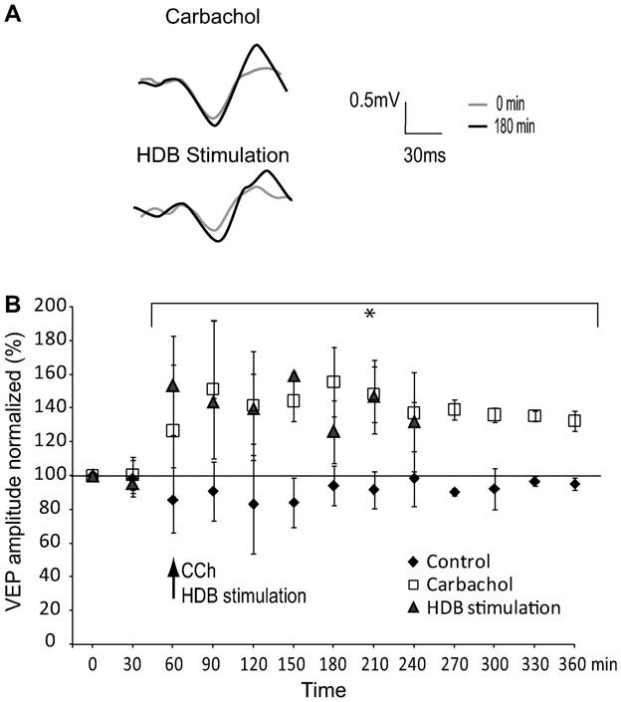
Effects of cholinergic system activation on VEPs amplitude. Cholinergic activation was performed through pharmacological injection or electrical stimulation paired with visual stimuli. (A) Representative wave of the VEP recorded before (grey line) and after (black line) CCh injection or HDB stimulation. The recorded wave was composed of a negative peak followed by a positive deviation representative of layer 4 field potentials trace. (B) Long-term effect on VEP amplitude of CCh infused in V1 (open square) or of HDB stimulation (triangle). After 2 periods of baseline recording (0 and 30 min), application of CCh or HDB stimulation (indicated by arrow) produces an increase of VEP amplitude observed for several hours after CCh infusion or HDB stimulation. Error bars indicate the SD values.

In our experimental conditions, the VEP was recorded as a wave composed of a negative peak followed by a positive deviation (Fig. 2) corresponding to electrophysiological signals recorded in cortical layer IV [19]. Mean amplitude difference between negative and positive peaks of the baseline VEP recorded was 0.965±0.08 mV. The amplitude (F_(7,70)_ = 1.915, p = 0.080) and the latency (F_(7,70)_ = 1.275, p = 0.113) of the VEP in the control animals did not change during the extent of the recording session (eight stimulations, 4 h, Fig. 2 and Table 1).

**Table 1:** Amplitude of VEP normalized after CCh injection or HDB stimulation and drug treatment.

Amplitude (%)	0 min	30 min	60 min	90 min	120 min	150 min	180 min	210 min
* Cholinergic enhancement*								
**Control**	100	99±11	86±19	91±17	83±29	84±15	94±12	92±11
**Carbachol (CCh)**	100	101±11	127±39*	151±41*	142±32*	144±12*	156±21*	149±16*
**HDB stimulation**	100	95±04	154±03*	144±03*	140±21*	159±02*	126±19*	147±22*
**Sco (i.p.)+CCh**	100	90±14	54±02*	89±14	78±15	86±19	95±18	114±08
**CCh+Sco (i.p.)**	100	95±08	130±45*	182±40*	165±17*	148±24*	162±04*	153±25*
* Pharmacological treatment*								
**aCSF+CCh**	100	95±15	105±13	92±12	115±18	122±14	130±18	112±13
**Sco (i.c.)+CCh**	100	78±26	108±34	93±24	64±05#	72±16#	84±29#	85±22#
**Mec+CCh**	100	99±10	115±10	101±10	77±21#	97±08#	90±09#	91±11#
**MLA+CCh**	100	102±06	94±07	83±15	152±29#	100±22#	86±06#	131±08#
**CPP+CCh**	100	106±22	91±08	111±16	88±14#	108±18	100±12#	81±15#

Values are expressed in mean±SD. For the first set of experiment (cholinergic enhancement) CCh infusion and HDB stimulation were administrated at t = 60 min. For pharmacological treatment, antagonists were injected at t = 60 min followed by CCh at t = 120 min.

* p<0.05, compared to control, ANOVA and LSD post-hoc.

# p<0.05, compared to aCSF+CCh, ANOVA and LSD post-hoc.

The mixed model ANOVA revealed a significant interaction of time and group in the amplitude (F_(21,182)_ = 10.505, p<0.001) between the control group and CCh injected, scopolamine injected (i.p.) and HDB stimulated group. One-way ANOVA at each time point revealed that a single injection of the cholinergic agent CCh paired with visual stimulation after stabilisation of the VEP (at t = 60 min), induced an increase (range 27–56%) in VEP amplitude that lasted for the whole period of stimulation (4 h) (LSD test, p<0.0001 compared to sham animals, Fig. 2). In the animals tested for a longer period of time (8 h), the enhanced effects were sustained for 6 h after which (remaining 2 h) there was variability amongst rats, probably due to the long-term isoflurane anaesthesia. The electrical stimulation of HDB paired with visual stimulation induced a long-term amplitude elevation of VEP (Fig. 2; Table 1), which was maintained during the whole period of time and was as great as CCh induced VEPs (compared to control group, p<0.001). There was no difference between the amplitude of VEPs induced by the CCh as compared to stimulation of HDB. Latency of VEP across the groups did not differ (Table 2, mixed model ANOVA, F_(21,182)_ = 1.429, p = 0.143).

**Table 2:** Latency of VEP after CCh injection or HDB stimulation and drug treatment.

Latency (ms)	0 min	30 min	60 min	90 min	120 min	150 min	180 min	210 min
* Cholinergic enhancement*								
**Control**	36±3	35±3	37±3	35±4	35±2	33±4	34±3	35±4
**Carbachol (CCh)**	37±3	37±2	36±4	34±3	29±3	26±5	31±3	31±3
**HDB stimulation**	35±4	32±2	33±3	33±3	35±3	33±4	36±1	35±3
**Sco (i.p.)+CCh**	37±1	41±1	40±2	40±2	35±4	38±3	39±5	37±2
**CCh+Sco (i.p.)**	38±1	38±3	35±1	33±1	33±1	35±5	33±3	36±1
* Pharmacological treatment*								
**aCSF+CCh**	38±2	37±4	35±4	33±3	36±3	32±3	32±3	35±2
**Sco(i.c.)+CCh**	38±5	37±2	41±1	39±3	43±5	40±6	44±5	39±6
**Mec+CCh**	39±1	38±3	38±2	37±6	46±3	45±4	30±4	38±4
**MLA+CCh**	40±2	41±4	39±2	37±4	36±4	37±3	39±3	37±2
**CPP+CCh**	39±3	36±4	36±2	34±3	38±2	38±5	31±4	30±6

Values are expressed in mean±SD of ms after the stimulation artefact. There was no effect of the treatment on latency.

### Effects of muscarinic, nicotinic and NMDA receptor inhibition on the amplitude enhancement of the VEPs

**Figure 3:**
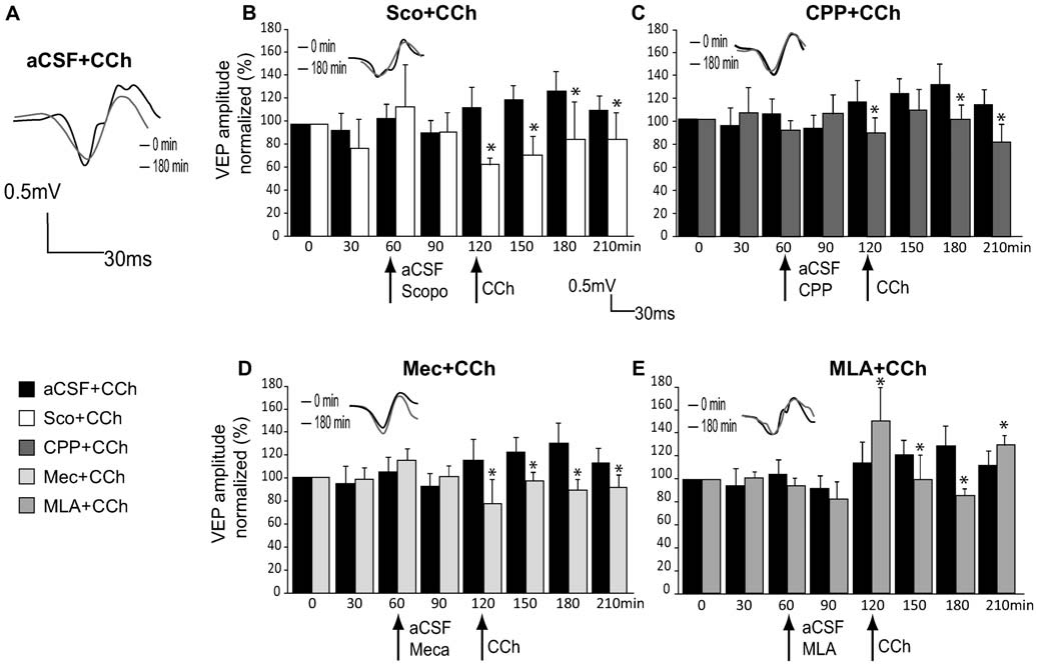
VEP amplitude changes after pharmacological infusion of drugs in V1. Effects of scopolamine (B, Sco+CCh), CPP (C, CPP+CCh), mecamylamine (D, Mec+CCh) or MLA (E, MLA+CCh) infusion prior to CCh administration are shown compared to aCSF+CCh injected animals as control group (black histograms). The long term enhancement of VEP amplitude is abolished in an identical manner by scopolamine, CPP and mecamylamine, suggesting that mAChR and nAChR could act upstream of NMDAR intracellular pathways. Drug infusion time points are indicated by black arrows.

The injection of inhibitors before the induction of CCh enhancement effect showed a significant interaction in amplitude between time and injected drugs (F_(28,168)_ = 7.979, p<0.001) but not in the latency (F_(28,168)_ = 1.105, p = 0.338). The amplitude of the basal VEPs (before infusion of CCh) was not affected by muscarinic (one-way ANOVA, p = 0.726), nicotinic (p = 0.236) and NMDA receptor inhibition (p = 0.115) during this administration nor 30 minutes after compared to the aCSF injected group. This suggests that none of the drugs injected contributed significantly to the baseline electrophysiological response to visual stimulation before CCh injection. Scopolamine (p<0.001), mecamylamine (p = 0.024) and CPP (p = 0.046) pre-treatment prevented the CCh-induced long-term enhancement of the amplitude of the VEPs (Fig. 3, Table 1). MLA showed fluctuating results (compared to the aCSF group values), that is, an increased VEP amplitude during CCh infusion (p = 0.003) and 2 h after CCh infusion (p = 0.02), but a decreased amplitude in between these two time points. The latency was unchanged for each group (Table 2, p = 0.086). VEP amplitude was however reduced (up to 32% decrease compared to control) at t = 120 min when CCh was infused in the scopolamine group (p = 0.028 i.c. and p = 0.048 i.p). Moreover, there was no effect of scopolamine (i.p.) when it was injected simultaneously with CCh (Table 1), suggesting that mAChRs do not contribute directly to the enhanced VEPs of CCh.

## Discussion

The principal objective of this study was to pharmacologically analyze the long-term effect of transient pairing of visual stimulation with cholinergic activation on cortical neuronal functioning. This was achieved by measuring changes in evoked potentials in V1 as a function of time. The results showed a long-term enhancement in the amplitude of the VEPs for at least 6 h when the cholinergic system was stimulated either from the cortex or the basal forebrain. This effect was mediated by different types of receptors, i.e. mAChRs and nAChRs as well as NMDARs but not α7 nAChRs. It is concluded that cholinergic agents induced LTP-like events in the cortex by triggering intracellular NMDAR pathways in glutamatergic cells. We discuss below the role of the cholinergic system in modulating cortical response to visual stimulation, its possible intracellular pathways and its relation to attention and learning processes.

### Acetylcholine modulates cortical responses in adult visual cortex

The results presented here demonstrate that a single synchronization between visual stimulation and cholinergic activation by CCh or electrical stimulation of the HDB was sufficient to induce a persistent increase of VEP amplitude lasting for several hours. Similar results were obtained by combining CCh injection and direct dorsal lateral geniculate nucleus tetanic stimulation in an LTP paradigm [21], and electrical basal forebrain stimulation combined with tactile stimulation [19], [30], [31]. As well, these results corroborate data obtained in cats, showing a long lasting response synchronization [8] or receptive field plasticity [10] of cortical visual cells after co-application of cholinergic agonists and light stimuli. These results confirm *in vivo* that long-term effects of visual stimulation are dependent on cholinergic activation. Interestingly, there was no spontaneous enhancement of VEPs amplitude in control conditions. This suggests that the low frequency visual stimulation in our experimental conditions did not increase ACh extracellular levels enough for inducing long-term effects in the control conditions. In agreement with this, it has been shown that visual stimulation with low frequency (0.067 Hz) checkerboards does not induce cortical long-term changes [32]. High frequency (9 Hz) stimulation does induce long-term changes in an effect termed sensory LTP [32], suggesting different neurobiological mechanisms involved in high and low frequency sensory stimulation.

### Involvement of NMDA receptors

The cessation of CCh-induced long-term enhancement of cortical response to visual stimulation during NMDAR inhibition supports the involvement of an interaction between cholinergic stimulation and NMDAR transmission [33], [34], [35]. The long-term enhancement of VEP reported here is similar to LTP mechanisms whereby synaptic strength is increased by the opening of NMDAR which launches a Ca^2^+ influx followed by an upregulation of glutamatergic receptors [36]. LTP occurrence is accompanied by an amplification of VEP [20], suggesting that the changes seen in the present study could reflect LTP.

The involvement of NMDAR is implicated in plasticity in the juvenile and adult visual cortex [37], [38] suggesting that NMDAR is a key factor in the plasticity induced by thalamocortical inputs. Although the occurrence of LTP peaks during the development period and drastically drops in the adult cortex, our results indicates that LTP-like mechanisms could participate in cortical plasticity in adult rats similar to what is reported in cat [39] and mouse [37]. Our results further implicate that these mechanisms are dependent on cholinergic mechanisms.

### Involvement of muscarinic receptors

Given that mAChRs are widely expressed in the visual cortex - the predominant postsynaptic mAChR being M1 subtype and the presynaptic mAChR being M2 [40] - and that M1 and M3 receptors are involved in hippocampal LTP [41], it was expected that inhibition of these receptors would abolish long-term enhancement of VEP. The present results of scopolamine administration verified this hypothesis since no long-term changes in VEP amplitude were seen after scopolamine infusion prior to CCh. This effect was robust and stable. Interestingly, i.p. infusion of scopolamine prior to CCh led to the same results as cortical infusion confirming that scopolamine i.p. could act at a local cortical target [21], [27], [42]. However, three findings suggest that mAChRs are involved in the induction of pathways generating long-term enhancement of electrophysiological responses, acting as a trigger mechanism rather than directly enhancing the ongoing neuronal excitability. First, the VEP amplitude was significantly decreased compared to baseline at the time of CCh infusion under scopolamine conditions which suggests that CCh may have a depressing effect during mAChRs antagonism. This effect might be mediated by nAChRs [16], [43], which could inhibit glutamatergic neurons through 1) activation of α4β2 or α7 nAChRs located on GABAergic neurons [14] or 2) disinhibition of inhibitory interneurons by blocking of M2 mAChR expressed by the GABAergic interneurons [44], [45]. Second, when mAChRs were fully inhibited secondary to CCh action (simultaneous scopolamine i.p. injection and CCh i.c. injection group, see methods), the enhanced long-term effects of CCh were not affected. This result suggests that mAChR activation is required for priming long-term enhancement of VEP but not directly for enhancing neuronal activity that contributes to the increase in amplitude of subsequent VEPs. This result contrasts with a recent study showing impairment of auditory memory when scopolamine was administered immediately after the cholinergic-paired training of the animal [42]. However, the electrical cortical responses were not recorded in this study, making it difficult to compare with our results. Finally, there was no significant difference between the effect of CPP and the one of i.c. scopolamine in terms of VEP amplitude. This might indicate an all-or-none effect on VEP enhancement, suggesting common intracellular pathways leading to LTP.

We propose that activation of mAChRs interact with intracellular NMDAR pathways to induce cholinergic-induced long-term effects on VEPs. It has been shown that M1 and M3 interact with NMDAR pathways in the hippocampus by elevating intracellular Ca^2+^ levels and thereby enhancing the AMPA receptor currents [46]. Post-synaptic mAChRs on pyramidal or spiny stellate cells are able to induce PKC or AKT [34], [35], which could be a mechanism of such intracellular interaction. Moreover, *in vitro* induction of LTP in V1 slices is impaired in M2/M4 mAChRs double knock-out mice [24], suggesting that inhibition of M2/M4 mAChRs impaired LTP. Alternatively, the long-term enhancement of VEP could result from an increase in VEP amplitude most likely due to the number and nature of cells involved or a change in the balance between LTP/LTD mechanisms induced. In this case, the inhibition of the different subtypes of mAChRs located on different cell types (GABA interneurons, pyramidal or spiny stellate cells) could result in a decreased number of excitatory cells activated by the paired visual stimulation and CCh infusion.

### Involvement of nicotinic receptors

Mecamylamine, a non-selective nAChRs antagonist, and MLA a selective α7 nAChR antagonist, were used to investigate the potential involvement of nAChRs in the long-term enhancement of VEPs. The α7 subtype of nAChRs is considered a key participant in cortical plasticity [14], but its potential role in the visual cortex has not been elucidated. Mecamylamine, but not MLA, showed an impairment of long-term increases of VEP. Results obtained with mecamylamine treatment were expected since it has been shown that its administration abolished LTP induced by tetanic stimulation of the dorsal geniculate nucleus in V1 [21], [22] and in V1 slice preparation [4]. These results have also been observed in sound-evoked cortical response in the auditory cortex [47]. Mecamylamine inhibits both α4β2 and α7 nAChRs that are located on thalamocortical terminals and cortical GABAergic neurons [14], [15], [48]. Activation of nAChRs located on the thalamocortical afferents increase thalamic input [7]. Inhibition of these receptors should result in the reduction of incoming signals from the thalamus which is in agreement with the abolishment of VEP amplitude enhancement under mecamylamine conditions in the current study. Inhibition of nAChRs located on the GABAergic cells may not be sufficient to explain these results since inhibition of these receptors should also result in reducing the inhibitory drive within the intracortical network, thereby lowering the threshold for eliciting a cortical response [6], [7], [17]. In addition, it has been shown that mecamylamine could transiently inhibit the NMDAR *in vitro* at the concentration used in the present study [49]. It is possible that the blockade of CCh-induced long-term effect on VEPs by mecamylamine in our study could result from an inhibition of the NMDAR located on the glutamatergic cells.

α7 nAChRs have been proposed to participate in cortical plasticity by activating silent AMPA receptors on glutamatergic neurons in the somatosensory cortex [17]. The blockade of α7 nAChRs in the present study did not consistently abolished the long-term enhancement of VEP induced by concomitant thalamocortical and cholinergic activation. The amplitude of the VEP response under MLA condition fluctuated, showing strong increases or decreases depending on the time point. This effect could be explained by an inactivation of GABAergic interneurons rather than glutamatergic cells during the α7 nAChRs blockade. Activation of α7 nAChRs of layer 1 interneurons has been shown to mediate disinhibition of cortical networks [15], which can result in increased VEP response. Consequently, inactivation of these receptors could generate decreases in VEP amplitude, whereas, increases in VEP amplitude could be induced by inhibition of GABAergic cells from layer 4. Such blockade of α7 nAChRs has been shown to induce LTP in the hippocampus [50], [51] due to their location on inhibitory interneurons [33].

### Functional implication of the cholinergic modulation of visual cortex

The permissive role by ACh shown here suggests that ACh is a key factor in experience-dependent plasticity allowing cholinergic enhanced stimuli to take over stimuli not associated with cholinergic reinforcement and modifying both cortical processing and representation of these stimuli. Our results bridge studies showing the role of the cholinergic system in selective attention (cholinergic reinforcement of visual stimuli) in V1 [1] and visual learning (long-term modification of synaptic responses and connections in V1) [2], [12], [52]. Our results imply that the cholinergic reinforcement of visual stimuli 1) would be provided by the adequately-timed cortical release of ACh from the basal forebrain terminals [27], [42] and 2) would be sufficient for visual learning [42]. These implications are further supported by previous work that ACh is released in cortex during numerous learning paradigms [12], [42], or visual stimulation [27], [53]. This release might be induced by sensory feed-forward influxes [27] or by top-down control, in which ACh mediates top-down attention mechanisms [1], [52] elicited by higher cognitive areas through basal forebrain activation [12], [54]. This interplay between stimulus driven and top-down input to modulate neuronal activity has been addressed by computational neurosciences [55]. In the computational model, the authors suggest that a reinforcement signal combined with an attention feedback signal, called attention-gated reinforcement learning, could model the cortical integration and mapping of sensory stimuli. The long-term mechanisms involving NMDAR and probably LTP pathways shown in the present study, suggest a modification of synaptic functioning by the cholinergic system, which would give a neurobiological basis to this attention-gated reinforcement learning. It would also suggest that attention and visual stimuli elicit ACh release in V1, which modifies synaptic functioning by eliciting LTP-like mechanisms at an early level of cortical processing.
